# The Fovea-Protective Impact of Double-Layer Sign in Eyes With Foveal-Sparing Geographic Atrophy and Age-Related Macular Degeneration

**DOI:** 10.1167/iovs.63.11.4

**Published:** 2022-10-06

**Authors:** Hisashi Fukuyama, Bonnie Bertha Huang, Ghazi BouGhanem, Amani A. Fawzi

**Affiliations:** 1 Department of Ophthalmology, Feinberg School of Medicine, Northwestern University, Chicago, Illinois, United States

**Keywords:** double-layer sign (DLS), geographic atrophy (GA), foveal sparing, age-related macular degeneration (AMD)

## Abstract

**Purpose:**

The purpose of this study was to investigate the impact of double-layer sign (DLS) on geographic atrophy (GA) progression in eyes with foveal-sparing GA and age-related macular degeneration (AMD).

**Methods:**

This is a retrospective, consecutive case series of eyes with foveal-sparing GA secondary to AMD with more than 6 months of follow-up. The size of the foveal-sparing area was measured on the fundus autofluorescence images at the first and last visits. Each eye was evaluated for the presence or absence of DLS inside the foveal-sparing area. We graded eyes based on the presence of DLS within the foveal-sparing area and compared the progression of GA between two groups (DLS (+) versus DLS (−)).

**Results:**

We identified 25 eyes with foveal-sparing GA with at least 2 follow-up visits (average interval = 22.7 ± 11.8 months between visits). The mean foveal sparing area was 1.74 ± 0.87 mm^2^ (range = 0.42–4.14 mm^2^) at baseline and 1.26 ± 0.75 mm^2^ (range = 0.25–2.92 mm^2^) at the last visit. Seventeen eyes (65.3%) were graded as DLS (+) within the foveal-sparing area. Square root progression of GA toward the fovea was significantly faster in the DLS (−) eyes (0.149 ± 0.078 mm/year) compared to the DLS (+) group (0.088 ± 0.052 mm/year; *P* = 0.04).

**Conclusions:**

The DLS (−) group showed significantly faster centripetal GA progression than the DLS (+) group. Our data suggest that the presence of DLS in the spared foveal area could be a protective factor against foveal progression of GA in eyes with AMD.

Age-related macular degeneration (AMD) is the leading cause of irreversible blindness in developed countries.^1^ Early and intermediate AMD eyes are characterized by drusen, whereas late AMD is defined by the development of either macular neovascularization (MNV) or the progressive atrophy of the photoreceptors, retinal pigment epithelium (RPE), and choriocapillaris, which is termed geographic atrophy (GA).^2^ Anywhere from 24 to 78% of eyes have multifocal GA, where the parafoveal lesions initially spare the fovea, enlarge over time, with ultimate coalescence of multifocal atrophic areas.^3^^,^^4^ A dramatic loss in^1^ central visual acuity occurs when the fovea finally becomes involved. As atrophy progresses, the GA lesions may form a partial ring surrounding the intact fovea, called foveal-sparing GA.^5^ The rate of progression of GA toward the fovea is a critical prognostic factor for vision in these patients.

Late-stage AMD is not always binary, with co-existent GA and MNV having a prevalence rate of 11 to 35% in AMD eyes on histology.^6^ Recent optical coherence tomography angiography (OCTA) studies have investigated the relationship between MNV and the rate of GA progression. One study found that the presence of either subclinical or exudative type 1 MNV was associated with reduced progression of atrophy in eyes with GA.^7^ Similar studies reported that subclinical MNV was associated with a slower GA growth rate.^8^^,^^9^ In contrast, Trivizki et al. found that subclinical MNV did not significantly influence local GA growth rates.^10^ Currently, anti-vascular endothelial growth factor (anti-VEGF) injection has shown great benefit in decreasing exudation and preserving vision in eyes with exudative MNV.^11^ Interestingly, the high-intensity anti-VEGF injection has been suggested to potentially increase the progression of GA.^12^ Hence, it seems that the presence of MNV, as well as its treatment, could influence GA, although these effects may have opposite directions, making this a very important area for research.

As a potential surrogate marker for subclinical MNV, double-layer sign (DLS) on optical coherence tomography (OCT) appears as an irregular, low-lying elevation of the RPE with low internal reflectivity. This sign has been observed in eyes with nonexudative AMD,^13^ and can identify subclinical MNV with good predictive values in these eyes (sensitivity 83–100% and specificity 74–94%).^14^^–^^16^

The purpose of this study is to investigate the impact of DLS on GA progression in AMD eyes with foveal-sparing GA. We hypothesize that the presence of DLS in the foveal-sparing area is a surrogate marker of subclinical MNV, which in turn could protect against GA progression toward the fovea. Investigating the impact of foveal subclinical MNV on the progression of GA has important implications in AMD eyes with foveal-sparing GA as it may suggest the need to avoid therapeutic approaches that aim for complete MNV regression in these eyes.

## Methods

This is a retrospective study of patients with AMD who have foveal sparing in GA at the Department of Ophthalmology at Northwestern University in Chicago, Illinois. The Institutional Review Board of Northwestern University approved this study, which followed the tenets of the Declaration of Helsinki. Data were extracted from the medical records. The ethics committee waived the requirement for written informed consent due to the retrospective and observational nature of the study.

### Participants

We reviewed the medical records of all patients diagnosed with AMD at Northwestern University from January 2016 to July 2021. The following inclusion criteria were applied: (1) GA secondary to AMD, (2) intact residual foveal island that was encircled by well-demarcated areas of GA involving more than 270 degrees of the circumference,^5^ (3) clear ocular media permitting good quality fundus imaging to analyze, (4) the interval follow-up with available imaging is more than 6 months; and (5) OCT, fundus autofluorescence (FAF), and near-infrared reflectance (NIR) at baseline and follow-up visits. Eyes treated with intravitreal injections, including anti-VEGF agents or any type of posterior-segment laser, including photodynamic therapy (PDT) during follow-up, were excluded from further analysis.

### Data Collection and Image Analysis

All patients underwent a standardized ophthalmologic examination, including measurement of visual acuity (VA), fundus examination, OCT, FAF, and NIR at the first and follow-up visits, as per the routine protocol in our center. OCT, FAF, and NIR reflectance images were acquired using a Spectralis HRA + OCT (Heidelberg Engineering, Heidelberg, Germany).

OCT volume scans and NIR images (820 nm) were acquired with a 30 degrees × 30 degrees field of view. FAF images were obtained with an excitation wavelength of 488 nm, an emission spectrum of 500 to 700 nm using the high-speed mode, and a field of view of 30 degrees × 30 degrees centered on the fovea with a resolution of 512 × 512 pixels.

### Image Analysis

**Figure 1.**
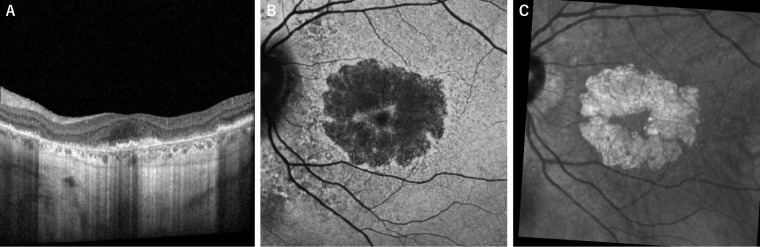
Multimodal imaging of foveal sparing in geographic atrophy: horizontal scan of spectral-domain optical coherence tomography (**A**), fundus autofluorescence imaging (**B**), and near-infrared reflectance imaging (**C**).

Each eye was evaluated for the presence or absence of DLS inside the foveal-sparing area by two independent graders (authors H.F. and B.B.H.). Disagreements were reconciled by discussion or by a senior third grader (author A.A.F.). We defined DLS +/− as the presence or absence respectively of an irregular low-lying elevation of the RPE from the underlying intact Bruch's membrane with low internal reflectivity of more than 250 um in the horizontal dimension and less than 100 um in height.^17^ We used FAF imaging to quantify the foveal-sparing region and GA area. The GA was characterized by severely reduced FAF signal due to the absence of fluorophores in the RPE in atrophic areas. For delineating the foveal sparing area, we used the NIR images in addition to the FAF to improve the accuracy of the measurements.^18^ The NIR reflectance signal increases in GA areas and is low in the foveal-sparing area.^19^ We used the NIR images for all cases in order to confirm the FAF delineation of the foveal-sparing and GA areas (Fig. 1).

Pixels were converted to mm^2^ using the formula:

$$\begin{array}{c} Area\;\left(mm^{2}\right)=\frac{8.85}{768}\;\times \;\frac{8.85}{768}\;\times \;pixel\;count\; \end{array}$$

**Figure 2.**
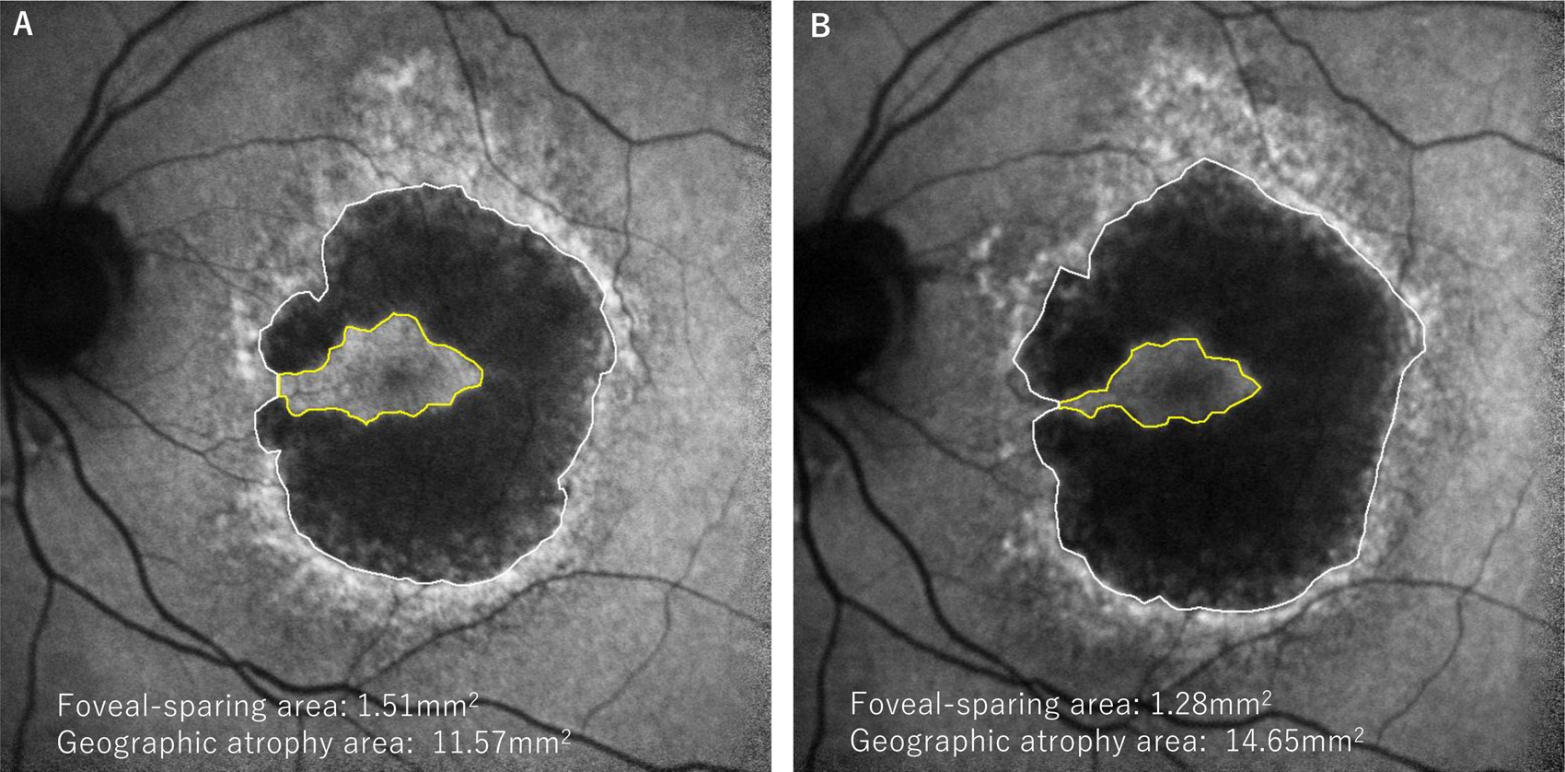
Measuring the fovea sparing area and geographic atrophy progression. The fundus autofluorescence (FAF) image at baseline (**A**) and the final, 20-month, follow-up visit (**B**).

Because the image resolution is 768 × 768 pixels and assuming the physical dimensions are 8.85 × 8.85 mm. Next, measurements of the foveal-sparing area and GA area were performed as previously described.^5^ The size of the foveal-sparing area and the GA area were measured on the FAF images at both the first and last visit (Fig. 2). In the case of incomplete foveal islands (the presence of bridges of intact RPE between the foveal-sparing area and the surrounding retina), the narrowest RPE bridges were used to demarcate the foveal-sparing region.^5^ Two independent graders (authors H.F. and B.B.H.) measured all the areas (foveal-sparing area and GA area) using the selection function of image J software (National Institutes of Health, Bethesda, MD, USA), and the average of the values obtained by the two graders was used as the final value. During measurement of GA and foveal sparing area, graders were masked to the presence of DLS on OCT.

The progression of GA was quantified in two directions. First, the centripetal progression of GA (atrophy spread toward the fovea) was calculated as the difference in the foveal-sparing area between the baseline and the last visit. Second, the centrifugal progression of GA (atrophy spread toward the periphery) was calculated by the difference between the baseline and last visit in GA area after subtracting the change in the foveal-sparing area. We transformed our data using the square root of the foveal-sparing area and GA to reduce the influence of the confounding relationship between baseline area and growth rates, as reported previously.^20^

Progression of GA was calculated using two metrics:

(1) Progression area rate (mm^2^/year), defined as
  $$\begin{array}{ccc} & & \mathrm{Centripetal}\;\mathrm{progression}\\ & & =\frac{Foveal-sparing\;area\;in\;the\;last\;visit\;\left(mm^{2}\right)\;-\;Foveal-sparing\;area\;in\;the\;first\;visit\;\left(mm^{2}\right)}{Last\;visit\;-\;First\;visit\;\left(years\right)}\\ & & \mathrm{Centrifugal}\;\mathrm{progression}\\ & & =\frac{\left(Foveal-sparing+GA\;areas\;in\;the\;last\;visit\;\left(mm^{2}\right)\right)\;-\left(Foveal-sparing+GA\;areas\;in\;the\;first\;visit\;\left(mm^{2}\right)\right)}{Last\;visit\;-\;First\;visit\;\left(years\right)} \end{array}$$
(2) Square root progression (mm/year)^21^ defined as
  $$\begin{array}{c} \mathrm{Centripetal}\;\mathrm{progression}=\frac{\sqrt{Foveal-sparing\;area\;in\;the\;last\;visit\;\left(mm^{2}\right)}\;-\;\sqrt{Foveal-sparing\;area\;in\;the\;first\;visit\;\left(mm^{2}\right)}}{Last\;visit\;-\;First\;visit\;\left(years\right)}\\ \mathrm{Centrifugal}\;\mathrm{progression}=\frac{\sqrt{\left(Foveal-sparing\;+\;GA\;areas\;in\;the\;last\;visit\;\left(mm^{2}\right)\right)}\;-\;\sqrt{\left(Foveal-sparing\;+\;GA\;areas\;in\;the\;first\;visit\;\left(mm^{2}\right)\right)}}{Last\;visit\;-\;First\;visit\;\left(years\right)} \end{array}$$

We compared the progression of GA using these two metrics between the two groups (DLS (+) versus DLS (−)).

### Statistical Analysis

All statistical analyses were performed using JMP Pro (version 15.2.0; SAS Institute Inc., Cary, NC, USA) and IBM SPSS Statics 28.0 (IBM SPSS Statistics Inc., Chicago, IL). VA was measured using a Snellen chart and then converted to logarithm of the minimum angle of resolution (log MAR) units for statistical analyses. Data were expressed as mean ± standard deviation. We used the Mann-Whitney *U* test tests or Fisher's exact test to compare the demographic characteristics and clinical characteristic, progression of area rate, and square root between two groups. Any *P* < 0.05 was considered statistically significant.

## Results

We originally identified 37 eyes from 28 patients with AMD who had foveal-sparing in GA. Of those, 11 eyes were excluded due to concurrent anti-VEGF injections during follow-up. Therefore, 26 eyes of 23 patients were selected for further analysis.

The Table shows the demographic and clinical characteristics of included patients. Of the 23 patients, 5 were men, and 18 were women. The mean age at the baseline visit was 84.3 ± 6.5 years. Twelve eyes were the right eyes, and 14 eyes were the left eyes. The mean follow-up interval was 22.7 ± 11.8 months (range = 6–38 months). The mean visual acuity (log MAR) at baseline was 0.34 ± 0.21 (range = 0–0.70) and 0.41 ± 0.18 at the last visit (range = 0–0.88).

**Table.** Demographic and Clinical Characteristics of Patients

Characteristics	Total	DLS (+)	DLS (−)	*P* Value
Patients	23	14	9	
Age, y	84.3 ± 6.5	85.2 ± 4.1	83.0 ± 9.3	0.53^a^
Gender, male (%)	5 (21.7)	3 (14.3)	3 (33.3)	0.28^b^
Smoking, current or former (%)	12	6 (42.9)	6 (66.7)	0.40^b^
Hypertension (%)	17 (73.9)	11 (78.6)	6 (66.7)	0.64^b^
Diabetes (%)	6 (26.1)	3 (21.4)	3 (33.3)	0.64^b^
Eyes	26	17	9	
Reticular pseudodrusen (%)	22 (84.6)	15 (88.2)	7 (77.8)	0.59^b^
Hyperreflective foci (%)	19 (73.1)	13 (76.5)	6 (66.7)	0.66^b^
GA in the fellow-eye (%)	24 (92.3)	15 (88.2)	9 (100)	0.53^b^
VA (Log MAR)				
Baseline	0.34 ± 0.21	0.36 ± 0.19	0.30 ± 0.23	0.36^a^
Last visit	0.41 ± 0.18	0.44 ± 0.14	0.37 ± 0.23	0.47^a^
Foveal sparing area, mm^2^				
Baseline	1.74 ± 0.87	1.67 ± 0.70	1.86 ± 1.15	0.69^a^
Last visit	1.26 ± 0.75	1.34 ± 0.70	1.09 ± 0.87	0.27^a^
GA area, mm^2^				
Baseline	8.40 ± 4.94	7.17 ± 2.40	11.0 ± 7.68	0.42^a^
Last visit	12.4 ± 6.8	110.6 ± 3.9	116.1 ± 9.9	0.20^a^

Abbreviations: GA, geographic atrophy, VA, visual acuity.

a Mann–Whitney *U* test.

b Fisher's exact test were used to calculate *P* values.

### Progression of GA

The mean size of the foveal-sparing area at baseline was 1.74 ± 0.87 mm^2^ (range = 0.27–4.14 mm^2^), and the mean GA area at baseline was 8.4 ± 4.9 mm^2^ (range = 2.77–25.1 mm^2^). The mean size of the foveal-sparing area at the last visit was 1.26 ± 0.75 mm^2^ (range = 0.15–2.95 mm^2^), and the mean GA area at the last visit was 12.4 ± 6.8 mm^2^ (range = 4.4–35.4 mm^2^). The intergrader agreement (ICC) was 0.959 (range = 0.930–0.977) for the size of the foveal-sparing area and 0.990 (range = 0.982–0.994) for the size of the GA area. Overall, centripetal GA progression rate was 0.26 ± 0.17 mm^2^/year (range = 0.04–0.65 mm^2^/year). Square root-transformation of data revealed centripetal GA progression rate of 0.109 ± 0.068 mm/year (range = 0.020–0.275 mm/year). Centrifugal progression rate was 2.13 ± 1.43 mm^2^/year (range = 0.57–6.3 mm^2^/year). After square root-transformation, centrifugal GA progression was 0.311 ± 0.176 mm/year (range = 0.090–0.841 mm/year).

### The Difference in the Progression Rate of GA Between Eyes With or Without Double-Layer Sign

**Figure 3.**
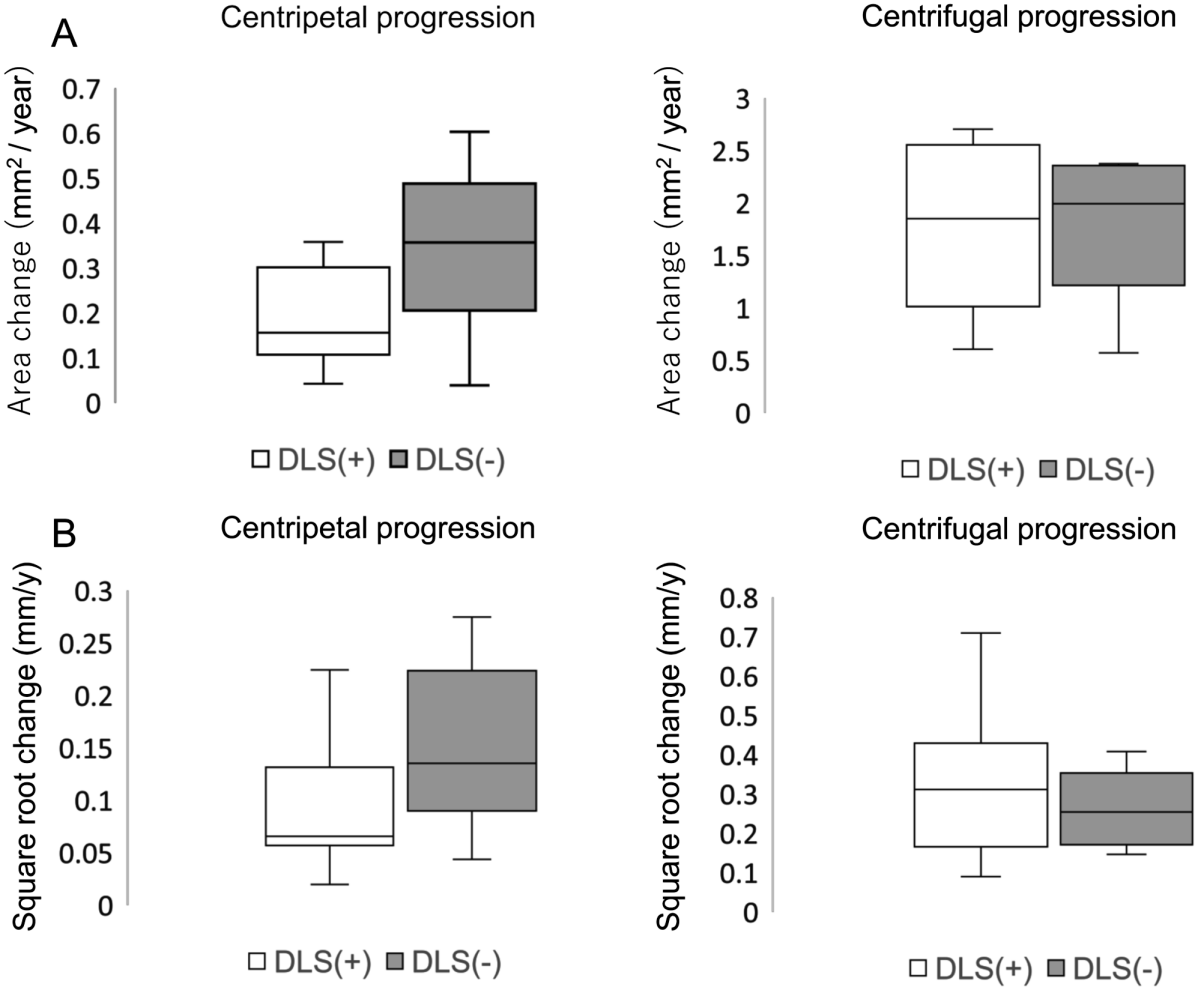
Box-and-whisker plots illustrate centrifugal (atrophy spread toward the fovea) and centripetal (atrophy spread toward the periphery) atrophy progression rates (**A**) and square root transformed progression (**B**).

Seventeen eyes (65.3%) showed a DLS inside the foveal-sparing area on OCT. The location of DLS was subfoveal lesions in 15 eyes and juxtafoveal in 2 eyes (Supplementary Fig. S1). Figure 3 shows the centripetal and centrifugal progression comparison between the two groups.

**Figure 4.**
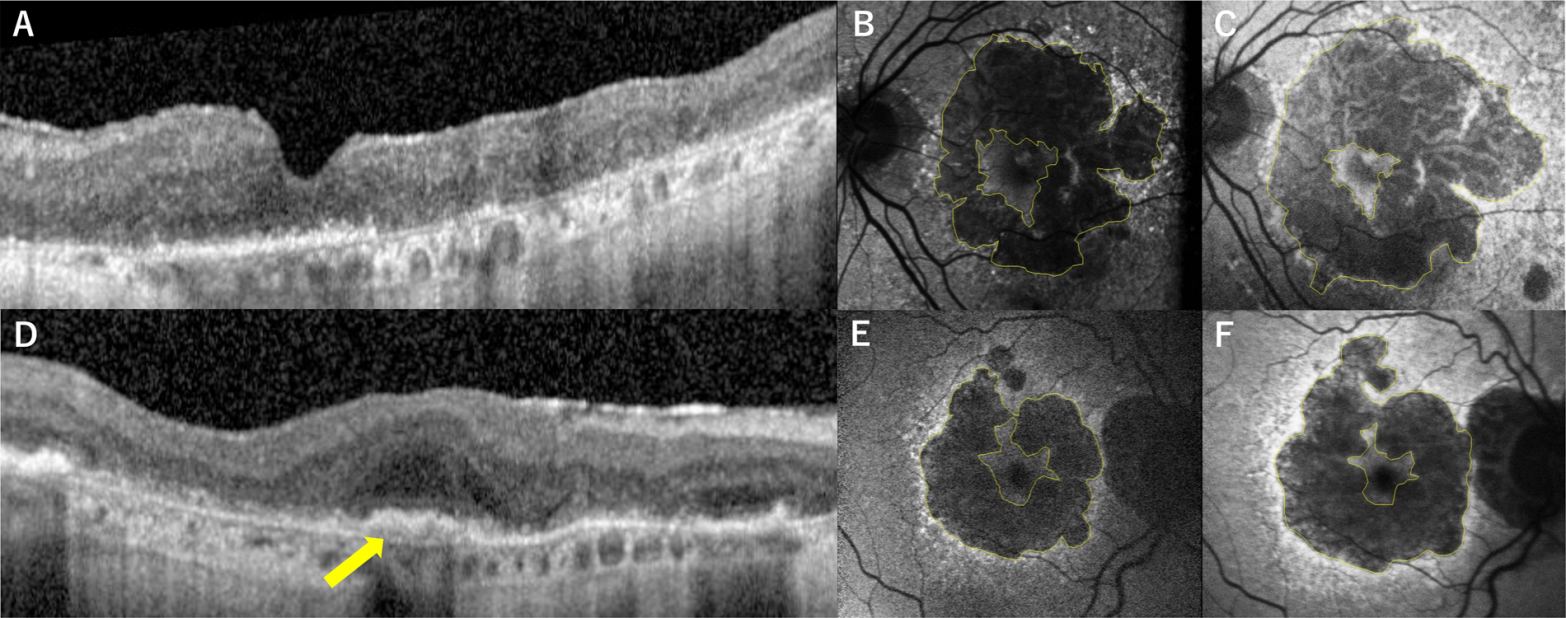
Geographic atrophy progression in eyes with or without a double-layer sign. Left eye of an 88-year-old female patient with geographic atrophy and foveal-sparing area. (**A–C**) OCT showed the absence of DLS **A**. Fundus autofluorescence (FAF) at baseline **B** and 25 months follow-up visit images showed 0.357 mm^2^/year GA progression toward the fovea. Right eye of an 87-year-old female patient (**D–F**) with geographic atrophy and foveal-sparing area. OCT structural B-scans showed DLS **D** (*yellow arrow*). FAF at baseline **E** and at 14 months follow-up visit images **F** showed 0.148 mm^2^/year GA progression toward the fovea.

The centripetal progression rate was 0.349 ± 0.186 mm^2^/year (range = 0.039-0.603 mm^2^/year) in the DLS (−) group and 0.214 ± 0.149 mm^2^/year (range = 0.043–0.654 mm/year) in the DLS (+) group (*P* = 0.07). Square root centripetal progression was 0.149 ± 0.078 mm/year (range = 0.044–0.275 mm/year) in the DLS (−) group and 0.088 ± 0.052 mm/year (range = 0.020–0.225 mm/year) in the DLS (+) group, which was significantly faster in the DLS (−) eyes (*P* = 0.04). The centrifugal progression rate was 2.05 ± 1.20 mm^2^/year (range = 0.57–4.59 mm^2^/year) in the DLS (−) group and 2.17 ± 1.56 mm^2^/year (range = 0.61–6.33 mm/year) in the DLS (+) group. Square root centrifugal progression was 0.268 ± 0.095 mm/year (range = 0.146–0.408 mm/year) in the DLS (−) group and 0.333 ± 0.203 mm/year (range = 0.090–0.841 mm/year) in the DLS (+), with no significant difference between the 2 groups (*P* = 0.77 and *P* = 0.68, respectively; Fig. 4).

## Discussion

In the present study, we compared the rate of progression of foveal sparing GA between eyes with and without DLS within the foveal-sparing region. Our study showed that the DLS (−) group had a significantly faster centripetal progression of GA than the DLS (+) group, suggesting a potential protective role of the foveal DLS. In our study, we used the structural OCT scan to identify DLS, which appears to be a clinically useful prognostic sign. GA progression into the fovea is significantly correlated with worse VA, making it an important clinical end point.^22^ Linder et al. reported 2.8-fold slower GA progression toward the fovea than toward the periphery (square root transformed rate = 0.116 vs. 0.319 mm/year, respectively),^5^ similar to the rates seen in our study (0.109 ± 0.068 mm/year vs. 0.311 ± 0.176 mm/year). Based on population-based cohort studies, foveal involvement in GA occurred after an average of 5.6 years.^23^ Therefore, identifying factors that could slow the rate of foveal involvement in GA would have great clinical and therapeutic value.

In this study, we considered the presence of DLS in the fovea-sparing area on OCT as a surrogate marker for co-existing subclinical MNV in eyes with GA (see Supplementary Fig. S1). Recent studies have shown that DLS on structural OCT was predictive of subclinical type 1 MNV, with the reported sensitivity and specificity between 83 to 100% and 74 to 94%, respectively.^14^^–^^16^ We therefore sought to evaluate the effect of DLS sign in foveal-sparing area on progression of GA toward the fovea, hypothesizing that submacular subclinical MNV would be protective. Indeed, our results suggest that submacular DLS could be considered a protective factor against the central progression of GA. On the other hand, the GA progression toward the periphery was not significantly different between the two groups. We hypothesize that DLS mainly influences the adjacent, local progression of GA and would therefore not expect central DLS to slow the progression of GA toward the periphery. Our results would support the idea that DLS has a limited local, and not a generalized, remote effect on GA, so that submacular DLS would not have an effect on peripheral GA progression.

Subclinical MNV has been proposed to have a protective effect against GA progression.^24^^,^^25^ Previous studies using OCTA investigated the association of subclinical MNV with the local progression of GA and suggested that subclinical MNV could have a protective effect against the progression of atrophy.^7^^,^^8^^,^^10^ Capuano et al. evaluated subclinical MNV using swept-source (SS)-OCTA in eyes with GA and found that the area spared from atrophy in had subclinical MNV in 13 out of 14 (92%) cases at the last follow-up.^8^ This was confirmed by Pfau et al. who showed that the progression of atrophy was markedly reduced focally in areas adjacent to subclinical or exudative type 1 MNV.^7^ These authors analyzed atrophy progression rates toward type 1 MNV, using local analysis and mixed-effects logistic regression, finding that the odds for future development of atrophy were significantly reduced by the presence of subclinical and/or exudative type 1 MNV. By contrast, Triviziki et al. used a GA growth model to estimate local growth rates and found that subclinical MNV lesions did inhibit adjacent local GA growth rates.^10^ Unlike our project, these previous reports did not focus on foveal MNV lesions and assessed correlations between local GA progression and subclinical MNV, rather than their foveal protective effects.

One of the proposed mechanisms for the protective effects of subclinical MNV suggests that capillary blood flow within the sub-RPE MNV lesion effectively compensates for the ischemic effects of underlying choriocapillaris breakdown secondary to AMD. Recent OCTA studies have shown that choriocapillaris flow deficit are increased around GA lesions,^26^ as well as a significant association between baseline choriocapillaris flow deficits and GA enlargement.^26^^,^^27^ Taken together, these studies would suggest that baseline choriocapillaris non-perfusion around GA lesions could be a risk factor for further GA progression. These findings also suggest that choriocapillaris loss at a distance may precede RPE changes in GA.^28^ In addition, the presence of subclinical MNV in AMD, by supplying oxygen to the hypoxic outer retina and RPE, may reduce oxidative stress, prevent oxidative damage, and potentially ameliorating RPE apoptosis.^29^

Although GA and MNV are frequently considered to be distinct subtypes of advanced AMD,^30^ histopathological and clinical studies have reported the co-existence of GA and MNV in the same eye.^6^^,^^31^^,^^32^ Whereas anti-VEGF treatment of active, exudative MNV is now standard of care, the role of anti-VEGF therapy in eyes with subclinical MNV is highly debated, which becomes even more complicated in eyes with co-existing GA. Molecular studies have shown that RPE-derived VEGF plays a key role in the maintenance of the choriocapillaris,^33^ and that loss of VEGF expression by the dysfunctional RPE layer in AMD could result in the atrophy of the underlying choriocapillaris.^34^ Thus, the protective effect of subclinical MNV may have several explanations suggesting that controlled, subclinical (non-exudative) sub-RPE neovasculature may be a desirable outcome in eyes with AMD, guarding against GA growth, especially in the fovea.^7^^,^^8^ Comparison of Age-Related Macular Degeneration Treatments Trials (CATT) have reported that monthly dosing of anti-VEGF had a higher hazard ratio for GA development than pro re nata injections.^35^ A recent meta-analysis similarly found an association between the frequency and number of treatments with anti-VEGF agents and the subsequent development of GA in neovascular AMD.^12^ In addition, the FLUID study demonstrated how treatment protocols that tolerated subretinal fluid (SRF, except for SRF >200 mm at the foveal center) could achieve similar visual acuity outcomes compared to treatments aimed at completely resolving all SRF.^36^ Based on these datasets, there is a growing concern among clinicians that aggressive anti-VEGF therapy may not contribute to visual acuity, whereas potentially having detrimental effects by enhancing the progression of GA.^12^

There have been no controlled clinical trials of anti-VEGF therapy in eyes with subclinical MNV without exudation. It is also important to note that although the presence of subclinical CNV portends a higher conversion to exudative disease (ranging from 20% to 80% over 6 months to 2 years of follow-up),^9^^,^^37^^,^^38^ the majority of these lesions continue to grow slowly without exudation.^39^^,^^40^ Furthermore, clinical trials of anti-VEGF prophylaxis in eyes with non-exudative AMD did not reduce the subsequent neovascular conversion rate.^41^^,^^42^ Taken together, the current clinical consensus is, therefore, to closely monitor subclinical MNV unless signs of exudation develop.^38^

We acknowledge there are some limitations to this study. A major limitation of this study was the small number of patients. The strict definition of foveal sparing GA identified a small number of patients in our practice. The small sample size also led to a smaller cohort considering the DLS (+) and DLS (−) groups. However, we found no significant differences in demographic and clinical characteristics including risk factors for progression of GA previously reported^43^^,^^44^ between the two groups. Another limitation was excluding the patients who developed exudative changes that required anti-VEGF injections during the follow-up period, so our study could not evaluate the DLS effect in this population. In addition, given its retrospective design, our study is susceptible to ascertainment bias. Whereas the sensitivity and specificity of DLS for detecting MNV are high (83–100% and 74–94%, respectively),^14^^–^^16^ the rates were reportedly slightly lower in late AMD compared to intermediate AMD in both (sensitivity of 74% vs. 90%, and specificity of 85% vs. 93%).^14^ Therefore, further studies using OCTA are needed to confirm the accuracy of DLS as a surrogate marker for subfoveal subclinical MNV in eyes with fovea-sparing GA.

In conclusion, our data suggest that the presence of DLS in the foveal sparing area may play a protective role against the foveal progression of GA in AMD eyes. In our study, the DLS (−) group showed significantly faster centripetal GA progression than the DLS (+) group. Further prospective studies are needed to determine the foveal protective effect of subclinical MNV in AMD eyes with foveal-sparing GA as well as the long-term course in these patients.

## Supplementary Material

Supplement 1
