# Supplementary material for: The Fovea-Protective Impact of Double-Layer Sign in Eyes With Foveal-Sparing Geographic Atrophy and Age-Related Macular Degeneration
Source: Invest Ophthalmol Vis Sci. 2022 Oct 6;63(11):4. doi: 10.1167/iovs.63.11.4 (PMC9554267; doi:10.1167/iovs.63.11.4)
Supplement: Supplement 1 [file iovs-63-11-4_s001.pdf]

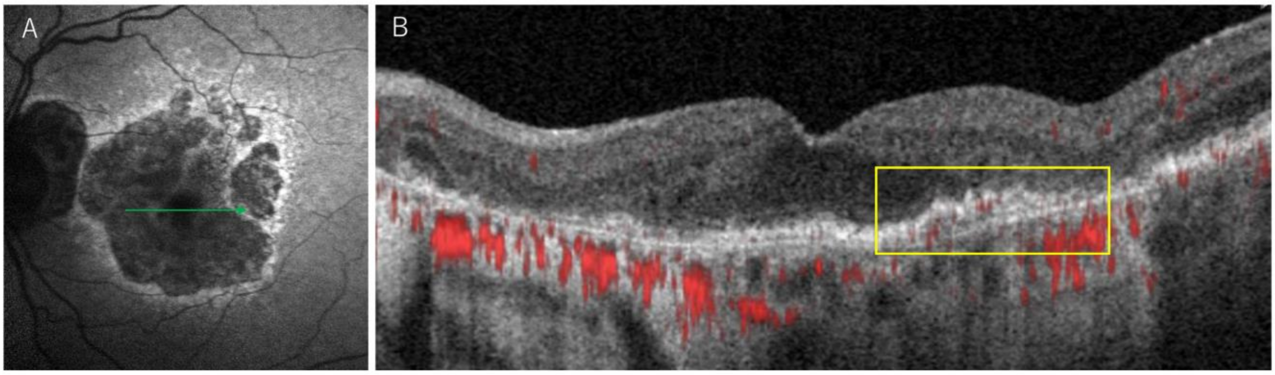

Supplemental Fig.1. Fundus autofluorescence (A) and B-scan OCT angiography image (B) finding in the eye with foveal sparing in geographic atrophy. OCT angiography showed the flow signal inside the juxtafoveal double-layer sign.
